# Transforming medical professionalism to fit changing health needs

**DOI:** 10.1186/1741-7015-7-64

**Published:** 2009-10-26

**Authors:** Thomas Plochg, Niek S Klazinga, Barbara Starfield

**Affiliations:** 1 Department of Social Medicine, Academic Medical Centre, University of Amsterdam, Meibergdreef 9, Amsterdam, The Netherlands; 2 Johns Hopkins Bloomberg School of Public Health, 615 N Wolfe Street, Baltimore, MD 21205, USA

## Abstract

**Background:**

The professional organization of medical work no longer reflects the changing health needs caused by the growing number of complex and chronically ill patients. Key stakeholders enforce coordination and remove power from the medical professions in order allow for these changes. However, it may also be necessary to initiate basic changes to way in which the medical professionals work in order to adapt to the changing health needs.

**Discussion:**

Medical leaders, supported by health policy makers, can consciously activate the self-regulatory capacity of medical professionalism in order to transform the medical profession and the related professional processes of care so that it can adapt to the changing health needs. In doing so, they would open up additional routes to the improvement of the health services system and to health improvement. This involves three consecutive steps: (1) defining and categorizing the health needs of the population; (2) reorganizing the specialty domains around the needs of population groups; (3) reorganizing the specialty domains by eliminating work that could be done by less educated personnel or by the patients themselves. We suggest seven strategies that are required in order to achieve this transformation.

**Summary:**

Changing medical professionalism to fit the changing health needs will not be easy. It will need strong leadership. But, if the medical world does not embark on this endeavour, good doctoring will become merely a bureaucratic and/or marketing exercise that obscures the ultimate goal of medicine which is to optimize the health of both individuals and the entire population.

## Background

There is a growing awareness that the current organization of medical work does not properly reflect the changing health needs. Organized medicine as we know it today is divided into numerous specialties and sub specialties [1]. This made sense when patients primarily suffered from single diseases that were treatable within the boundaries of one (sub) specialty. However, it is dysfunctional when a growing number of patients suffer from chronic and overlapping health problems (multi-morbidity) and this is illustrated by the increasing problems related to cost, the workforce and the quality of the work [2-5]. A high degree of skilful coordination is often promoted as the ideal remedy [6,7], but a basic re-organization of the way that the medical system works is needed in order to adapt to today's changing health needs.

Although the organization of medicine is beginning to reflect changing population's health needs, progress appears to be minimal and slow. Relatively new specialties (for example, geriatric medicine and critical care medicine) provide medical expertise organized around the health needs of older people and critically ill patients as family practices have done for its patients. However, physicians trained in these fields do not appear to be leading a charge for system reform and probably cannot do so given the reality of professional power of the vested interests of other specialties [8].

Arguably, a health policy could trigger support and speed up the needed changes; the sociological literature on professionalism provides clues as to how this might be done [9,10]. Research evidence shows that purposefully calibrated policy pressures can trigger effective self-regulation. For instance, policy imperatives were crucial in the 'compulsory' introduction of medical audit in England and The Netherlands [11]. Similarly, drawing medical specialists into management is often put forward as a strategy to improve hospital management [12-14]. However, key stakeholders opt to enforce coordination and remove power from the medical profession when they are planning improvements in the performance of the health services system. This is illustrated by proposals such as the 'chronic care model' and 'system-wide redesign', which essentially promote non-physician coordinators, multidisciplinary teamwork and integrated care arrangements, and leave the splintered organization of the medical world intact [15-17]. This paper proposes a long-term vision accompanied by several strategies by which the self-regulatory capacity of the medical profession is consciously activated in order to transform the medical profession and its related professional processes of care into a system that can deal more efficiently with the changing health needs.

## Discussion

### Professionalization in medicine

Professions are defined as groups of institutions that permit the members of an occupation to make a living while controlling their own work [18]. Internal-control is a basic characteristic of professions, as they perform knowledge-based work that is inaccessible to those lacking the required training and experience, and that cannot be standardized. In other words, the status of a profession is linked to work that cannot be controlled other than by the work force itself.

As the status of medical work is, at least in part, competitive, professionalization is linked to the pursuit of internal control over medical work and outperforming rival specialties. Specialty boards have to demonstrate the superiority, exclusiveness and discretionary nature of the knowledge which underpins their work. Meeting this requirement is a huge endeavour, as jurisdiction over medical knowledge cannot be claimed by decree alone, particularly in an era of evidence-based medicine. It must be established alongside, or at the expense of, other specialties with a vested interest. Thus, turf battles are inherent to the professionalization processes even when medical specialties are interdependent and form an alliance or so called 'system of professions' [19].

When knowledge becomes very complex, specialization in just one segment of it makes the work more manageable by limiting breadth while permitting depth and innovation. In the medical world, this traditional way of reducing complexity is based on the assumption that the human body can be reduced to smaller and simpler components, and that understanding each component separately leads to an understanding of the entire health problem - that is, that the whole is the sum of the components [20,21]. Under this reductionist assumption, innovation in medical science results in knowledge on smaller and smaller bodily parts reflected by an ever growing number of deeper and narrower (sub) specialties.

An alternative assumption is that the whole is more than the sum of its parts. Fields such as geriatrics, critical care medicine and family medicine build upon the recognition that diagnosis and treatment require the generalist focus on 'bodily systems' rather than the specialty focus on 'bodily organs' - a notion consistent with the upsurge of 'systems thinking' in medical science: human beings are viewed as composed of and operating within multiple interacting and self adjusting systems (including biochemical, cellular, physiological, psychological, and social systems) [21-23]. In a systems approach, a complex health problem is made manageable by observing the overall pattern in the behaviour of the variety and interactions of bodily systems. Medical innovations based on 'systems thinking' would result in more generalist knowledge reflected by stronger primary care infrastructures and/or more generalist specialty domains in secondary and tertiary care.

### Dysfunctional results of current professional processes

Both views, the one leaning toward specialization and the other towards generalization, provide valid and valuable medical knowledge. Thus far, medical professionalization processes have, for the most part, been driven by medical innovations based upon the former. However, it seems worthwhile to stimulate innovations based upon the latter as they may indicate a potential way to obtain a configuration of medical specialties that better fits the changing population health needs [21].

Evidence shows that chronic diseases are highly endemic, fast becoming the major type of morbidity in industrialized countries [24,25]. This pattern, known as the fourth stage of epidemiological transition, presents new realities in the understanding of the genesis, manifestation and prognosis of health problems [26]. A chronic disease is one that is likely to recur, persist and last for more than one year. Typically, these diseases have multifactorial causes involving risk factors and preclinical conditions that usually are not amendable to reversal. As a consequence, people with chronic diseases tend to have multiple, overlapping and long lasting health problems [27].

The existing configuration of medical specialties and related professional processes of care poorly address this new morbidity pattern. Having multiple chronic, complex and overlapping health problems is associated with poor outcomes in terms of quality of life, psychological distress, longer hospital stays, more postoperative complications, higher mortality and higher costs of care [2,6,28]. These are, at least in part, attributable to the splintered and overly specialized professional processes of care that are still based upon morbidity patterns typical for the previous stage of epidemiological transition (that is, single diseases with unitary causes and of relatively short duration). Chronically ill patients consult multiple medical specialists in a year working across different settings whose inputs are poorly reconciled and poorly coordinated [2,29].

Primary care physicians should have the expertise, knowledge and competence to consistently coordinate all the inputs from various doctors and navigate patients through the system. Countries with a strong primary care infrastructure have better outcomes in terms of population health, costs, access and coordination experiences [30,31]. Logically, the strengthening of primary care is widely considered to be an indispensible feature of well performing health care systems in the twenty first century [32].

Nonetheless, it is naive to believe that the strengthening of primary care alone will adapt the medical profession's practices to the changing health needs. Primary care physicians would still encounter problems in performing their tasks and organized medicine would remain fundamentally fragmented [33]. Moreover, building strong primary care infrastructures without making changes in number of physicians in secondary and tertiary care will require the production of a larger physician workforce. This would be unaffordable and/or it would be necessary to import physicians from low income countries: an unethical solution [34-36].

There is little evidence that current proposals for care, such as the Chronic Care Model, are measurably improving patient care, even of those with chronic illness [37,38]. Although considerable progress has been made in making the medical work place more accessible to patients and making it eligible for control by non-physician managers, the nature of the work still requires a considerable degree of tacit, discretionary and experiential expertise, an inherent aspect of professional work. Well performing healthcare systems in the twenty first century need doctors who retain their professional roles and also remain the key protectors of quality. The route to improving health care delivery for those suffering from chronic disease patterns is not by-passing and curtailing medical professionalism but, rather, to establishing more generalist specialties alongside the existing ones.

### Towards a new configuration of medical specialties

To achieve a new configuration of more efficient medical specialties, while freeing up resources for more generalist specialties, three interrelated steps are proposed. The first entails defining and categorizing populations according their burdens of morbidity. New categories are needed in order to classify patients with multiple, recurrent and long-lasting health problems that provide the basis for gathering and organizing medical expertise [27,39]. For example, what expertise is needed to deliver optimal medical care to patients with multi organ disorders or a frail elder with diabetes and heart failure? There are categorizations that explicitly aim to characterize the degree of total morbidity burden from a clinical and epidemiological perspective, (see ). Moreover, intensive care medicine, paediatrics, occupational medicine, emergency medicine and geriatrics mark fields in medicine where more generalist specialties would be advantageous. Nevertheless, which categories will ultimately be formed will depend on a study of the potential of the different alternatives to deal with multi-morbidity. Research on this theme and related issues is still in its infancy [27].

The second step requires that professional work be organized around the newly defined and categorized health needs. This essentially means merging or rearranging specialty domains or establishing new ones. For example, one may conclude that geriatrics should be established as a fully approved medical specialty, thus making geriatricians the frontline staff for frail elderly patients [8,40]. This must then be reconciled with the prerogatives of existing medical specialties, such as internal medicine, cardiology and neurology. More important, expanding the number of generalist specialties may blur the interface between primary, secondary and tertiary care services. This would have far reaching consequences for the staged organization of health care systems, in particular the 'gate' function of primary care. It may turn out that locating one central coordinator or navigator role in primary care is less useful than juxtaposing fields such as paediatrics, occupational medicine, geriatrics and emergency medicine next to general practice. A rearrangement of specialty domains is unlikely to occur by decree; broader specialty domains will have to be established from within, strategically supported and stimulated from the outside and based on a vision of health system design with special reference to the blurring of the interfaces between primary, secondary and tertiary care for people with multi-morbidity.

The third step is to reorganize the work of doctors practicing these broader specialty domains. A major challenge will be to devolve tasks and responsibilities to the type of physician most accessible to patients and consistent with the achievement of excellent quality and outcomes. Aggravating an already full workload is a likely outcome. Therefore, professional work that is non-discretionary in nature and which therefore can be standardized or managerially organized will have to be devolved to less highly educated health personnel. This is better known as 'task substitution', for which there is well-established literature that illustrates the potential and feasibility of transferring tasks to non-physicians [41]. Moreover, tasks can also be left to the patients themselves - with backup from the professionals - as illustrated by the developments in telemedicine and self care [42-44].

### Policy implications

The challenge of achieving the proposed new configuration of medical specialties is daunting. It will run counter to the existing *status quo*, as it rearranges specialty domains, resources and incomes. This creates winners and losers and one can expect prospective losers to oppose such change. Nevertheless, the basic strategy for change is straightforward: substitute a person and population health-focused view for an organ or disease-focused one. That is, the categorization of people according to their burdens of morbidity will allow not only a more rational way of stratifying the population according to their degree of need, but it will also facilitate the identification of population subgroups that are especially vulnerable and may profit from more generalist domains and related professional processes of care. By making such an orientation more prestigious, rewarding and beneficial than an organ or disease orientation, the configuration of medical specialties, as described by Abbott, could more easily evolve [19].

The critical challenge is to promote and strengthen such an orientation in practice and enable it to become mainstream. This requires medical leadership, as the medical profession itself is largely responsible for the way in which medical expertise is organized. Medical professions must recognize that the proposed long-term vision is a promising route towards both improving performance in healthcare and protecting the values and principles of medical professionalism against the countervailing forces of the free market and bureaucracy [45,46]. The likelihood that professions will take up this endeavour could be increased by a set of well-calibrated external policy pressures in at least seven areas (Table 1).

**Table 1:** Strategies

**Strategy**	**Description**
Defining medical professionalism	Promoting a population health orientation as one of the core values of medical professionalism.

Create supportive professional bodies	Easing the requirements that emerging specialties need to satisfy in order to become a fully approved medical specialty board.

Targeted research funding	Establishing an enhanced portfolio of medical research that provides the credentials for more generalist medical specialty boards.

Targeted technology development	Investing in the development of new technologies that favour generalization rather than (sub) specialization.

Modernization of medical curricula	Including expert decision making based on the principles of systems thinking and multi-morbidity in medical education.

Performance management	Developing performance based instruments related to the health outcomes of the patient groups that are served rather than for individual diseases.

Supportive payment models	Developing pay-for-population-health-performance schemes that reward medical professionals for maximizing population health outcomes.

First, an explicit population health orientation should be one of the core values of medical professionalism. Much has been accomplished in the definition, measurement and the inculcation of core professional values. For instance, the working group of the Royal College of Physicians highlights 'integrity', 'compassion', 'altruism', 'continuous improvement', 'excellence' and 'working in partnership with the wider healthcare team' as values underlying the practice and science of medicine [47]. In order to stimulate the desired effect, this list should explicitly include a population health orientation through which medicine, acting as a collective, agrees to consider its contribution to society in relation to its initial purpose (that is, to maximize health for both the individual and the population as a whole). It would mean that organized medicine agrees to operate within the constraints of a vision that sets the conditions deriving from the health situation of the population, or community, it is designed to serve, which resonates with the call for 'civic professionalism' [48].

Second, the current procedures for approving new medical specialties favour specialization rather than generalization. With a more efficient use of current specialists, resources could be freed up for the development of new and more generalists' domains to meet the needs of specific populations - a process that has been difficult to achieve because of the existing procedural barriers which hinder the demarcation of new generalist expertise [49]. Emerging specialties have to demonstrate that they represent a well-defined field of medical practice in their own right [50]. This requirement will be especially hard to satisfy for the proposed generalist domains, since their underlying knowledge will be broad and will overlap with the vested ones. The absence of a clear definition of the field may explain the relatively low prestige of branches of medicine such as geriatrics, intensive care and emergency medicine. Existing professional procedures frustrate the opportunity for such specialties to build their own professional bodies and colleges.

Third, an enhanced portfolio of medical research could further the person-orientation of medical care by examining whether, and how, more generalist domains deliver the hypothesized benefits using a systems paradigm [21,22]. As the research infrastructure of academic medicine largely coincides with current vested specialties, a research endeavour to provide the credentials for the transformation of medicine proposed in this paper will need special targeted funding.

Fourth, investments in technology should be directed towards those innovations that support generalization rather than specialization. In particular, the development of telemedicine has the potential to further the recognition of people- and population-focused care [44]. Current forecasts reflect the further miniaturization of medical devices, replacement organs and tissue engineering advances, molecular and gene-based diagnostics. Doctors and manufacturers closely collaborate allowing vested medical specialties to move technology forward in the direction that suits their professional interests.

Fifth, medical education needs to be modernized in additional directions to those commonly advocated, such as expanding the generalist workforce or transforming it towards the Chronic Care Model [51,52]. The focus of this modernization should not only be on learning non-clinical competencies (for example, enhancing self-management by patients, teamwork, application of quality instruments and management) but, rather, on learning expert decision-making based on the principles of systems thinking, including multi-morbidity. These new competencies should be applied to the care of all people, not only those with specific chronic conditions.

Sixth, the instruments needed to maintain and improve the competence of medical doctors throughout their careers must become performance-based and related to the health (rather than the disease) outcomes of the patient groups that they serve. The 'renewing medical professionalism debate' needs to be linked to the broader debates on quality improvement and health systems performance. The yield of both debates, in terms of developed frameworks and instruments for performance improvement, should be reconciled. While the debate has yielded profession-owned instruments to maintain and improve individual performance of doctors, the quality of care debate is now moving ahead, evaluating healthcare system performance in relation to population health [53].

Lastly, payment models need to be developed in order to engage doctors in the reorganizing of medicine. Despite the efforts to launch value-based purchasing and pay-for-performance models, few health are systems have succeeded in putting in place payment models that go beyond the goals of controlling costs and rewarding the providers who achieve selected performance benchmarks [54]. Proposals to move pay-for-performance towards pay-for-population-health-performance have already been suggested [55]. Paradoxically, this move might turn out to be more fertile than just pay-for-performance. Physicians would probably have a strong interest in reorganizing their medical work in the proposed direction, if their rewards are linked to their performance in terms of population health outcomes. Apart from huge technical challenges, such a pay-for-population-health-performance scheme essentially aligns the interest of society and the professionals, which will support the proposed transformation of medical professionalism.

## Summary

The deliberations of organized medicine have not kept pace with the changing health needs and potential strategies for management. Few promote the reorganization of the medical professions as a potential solution to new and emerging needs. Rather, the ongoing (sub) specialization within medicine and its splintering effect on health care delivery seems to be an accepted imperative. This paper challenges this idea. It argues that medical professionalism can, and should, be reoriented in the face of ageing populations increasingly suffering from complex and chronic diseases, accelerating costs and the inevitable health workforce crisis.

By instilling in the medical profession the belief that population health needs should be the leading principle for the professionalization processes within medicine, professional models of care could be transformed in such a way that complex and/or chronically ill patient populations are better served. This transformation features three consecutive steps: (1) defining and categorizing the population health needs; (2) reorganizing specialty domains around the needs of populations with specific needs; and (3) reorganizing specialty domains by eliminating work that could be done in primary care or by the patients themselves.

Taking this alternative road towards health care improvement will not be easy. It calls for strong leadership in all the medical professions. But, if medicine does not embark in this endeavour, good doctoring will become merely a bureaucratic and/or marketing exercise that obscures the ultimate goal of medicine - optimizing the health of both individuals and the population.

## Competing interests

The authors declare that they have no competing interests.

## Authors' contributions

TP initiated the project, developed the ideas and drafted the manuscript. NSK and BS helped to elaborate on the ideas and to draft the manuscript.

## Pre-publication history

The pre-publication history for this paper can be accessed here:


